# Supplementary material for: Understanding Changes in the Topology and Geometry of Financial Market Correlations during a Market Crash
Source: Entropy (Basel). 2021 Sep 14;23(9):1211. doi: 10.3390/e23091211 (PMC8467365; doi:10.3390/e23091211)
Supplement: Supplementary file 1 [file entropy-23-01211-s001.zip › entropy-1326084-supplementary.pdf]

A1

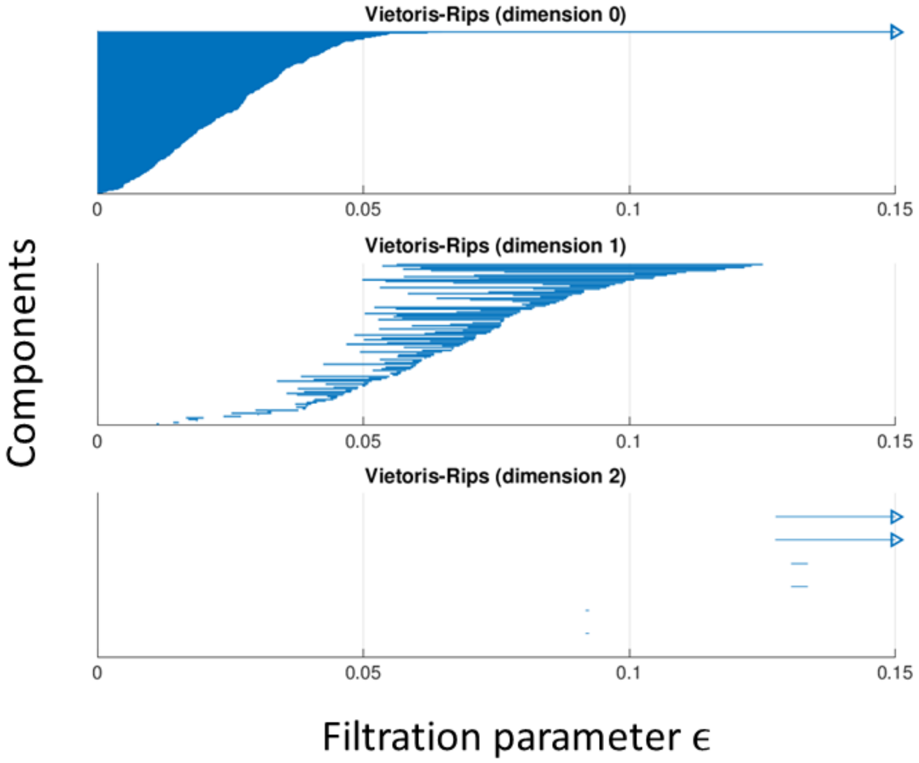

Vietoris-Rips (dimension 0)
0
0.05
0.1
0.15
Vietoris-Rips (dimension 1)
0
0.05
0.1
0.15
Vietoris-Rips (dimension 2)
0
0.05
0.1
0.15
Components
Filtration parameter ϵ

A2

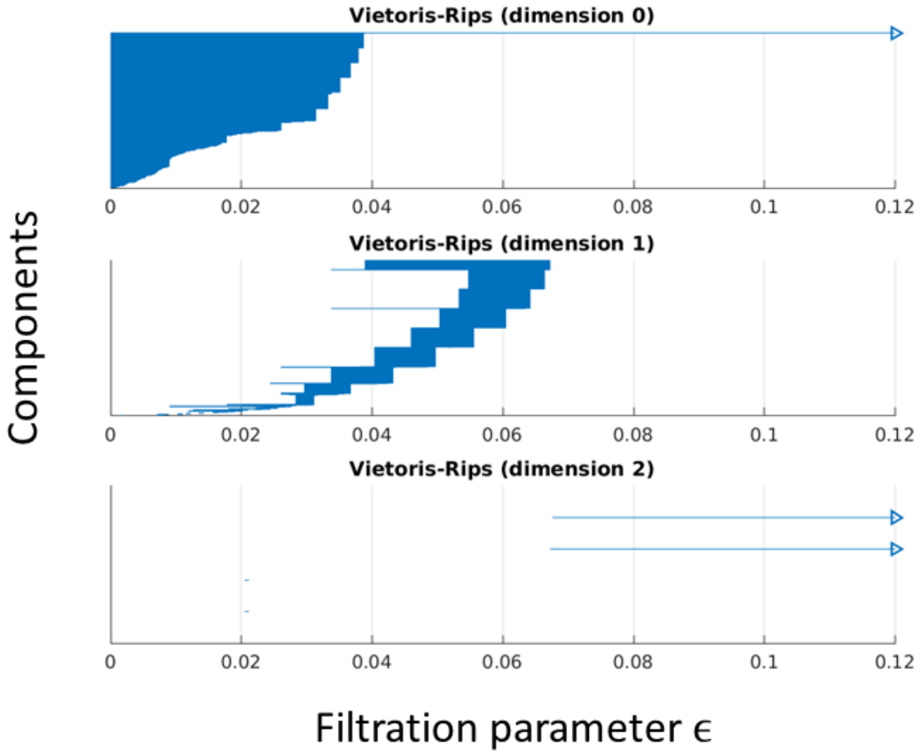

Vietoris-Rips (dimension 0)
0
0.02
0.04
0.06
0.08
0.1
0.12
Vietoris-Rips (dimension 1)
0
0.02
0.04
0.06
0.08
0.1
0.12
Vietoris-Rips (dimension 2)
0
0.02
0.04
0.06
0.08
0.1
0.12
Components
Filtration parameter ϵ

A3

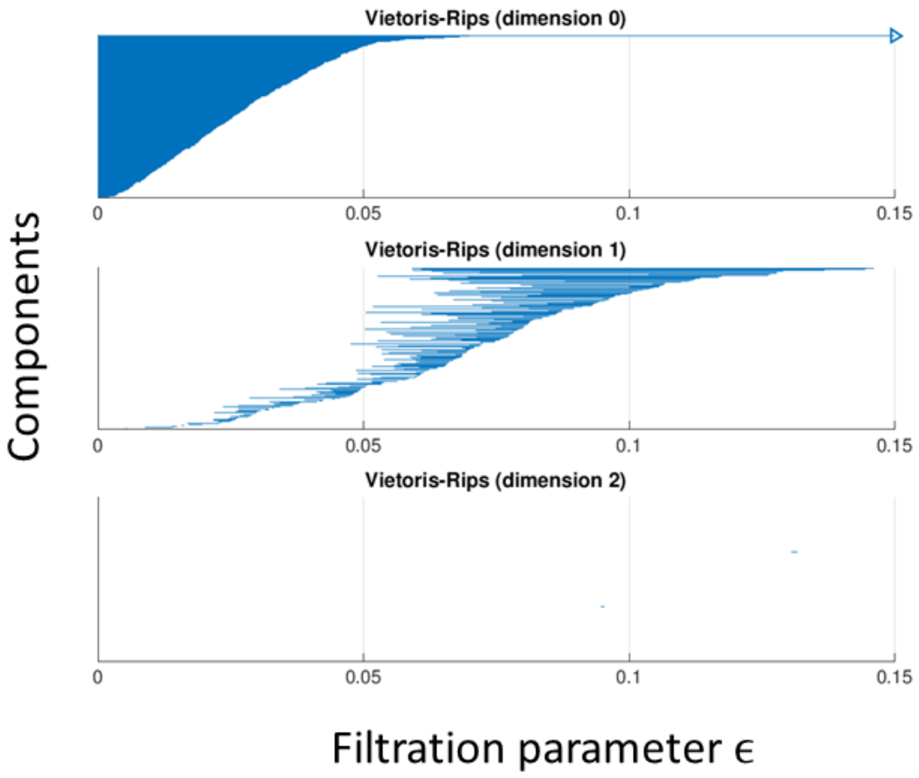
Vietoris-Rips (dimension 0)
0
0.05
0.1
0.15
Vietoris-Rips (dimension 1)
0
0.05
0.1
0.15
Vietoris-Rips (dimension 2)
0
0.05
0.1
0.15
Components
Filtration parameter ϵ

A4

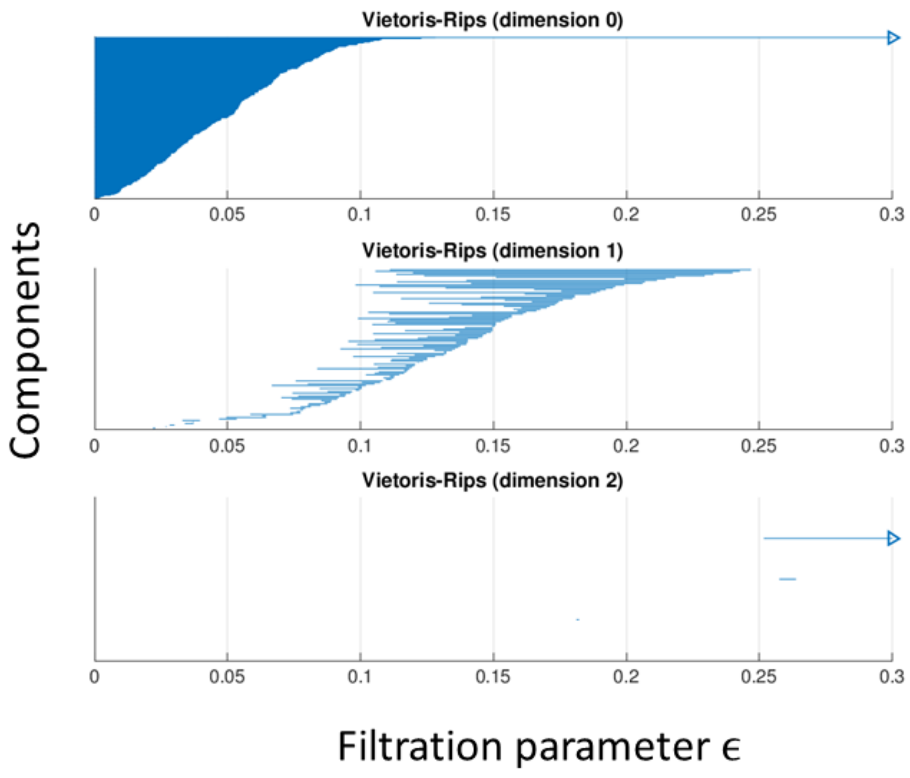
Vietoris-Rips (dimension 0)
0
0.05
0.1
0.15
0.2
0.25
0.3
Vietoris-Rips (dimension 1)
0
0.05
0.1
0.15
0.2
0.25
0.3
Vietoris-Rips (dimension 2)
0
0.05
0.1
0.15
0.2
0.25
0.3
Components
Filtration parameter ϵ

B1

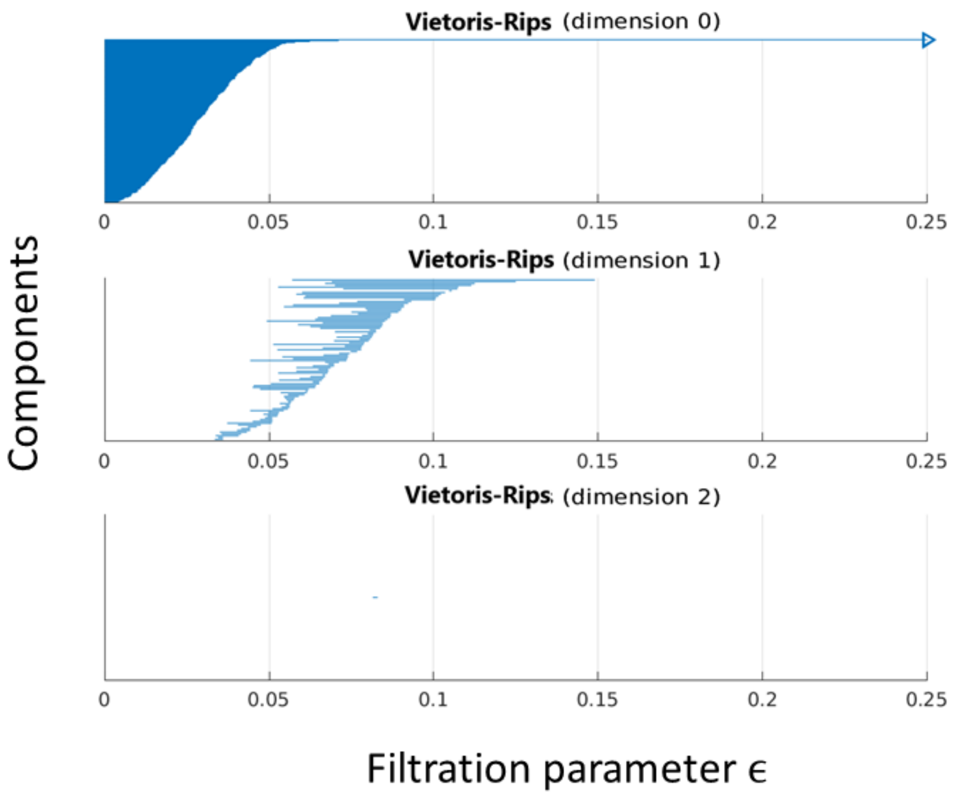
Vietoris-Rips (dimension 0)
0
0.05
0.1
0.15
0.2
0.25
Vietoris-Rips (dimension 1)
Vietoris-Rips (dimension 2)
Components
Filtration parameter ϵ

B2

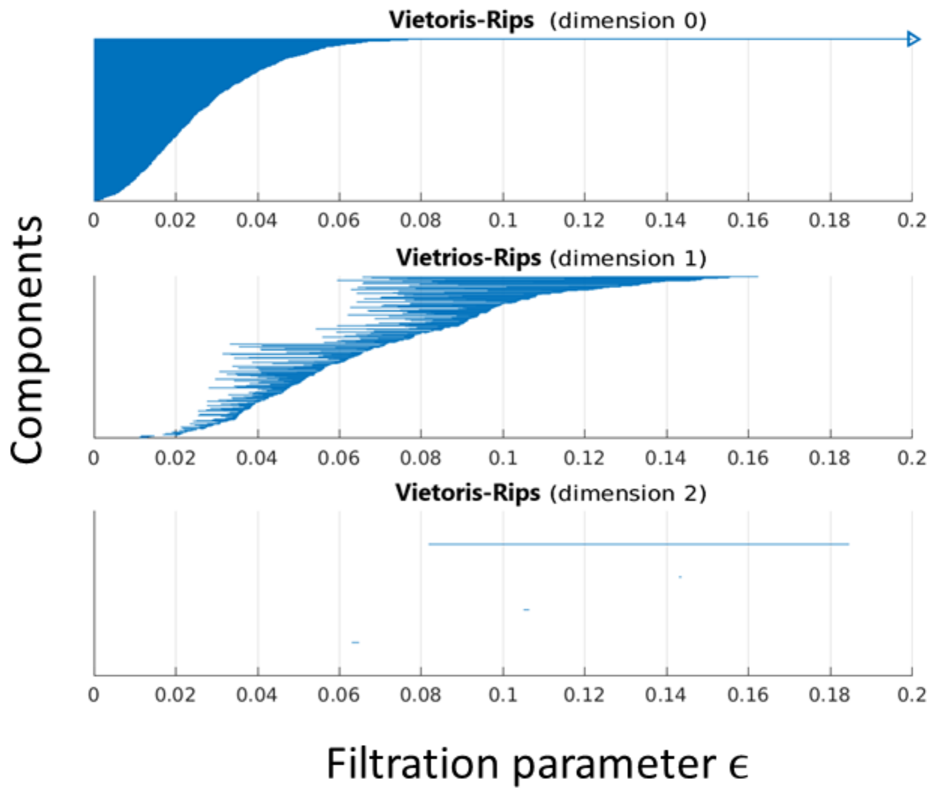
Vietoris-Rips (dimension 0)
0
0.02
0.04
0.06
0.08
0.1
0.12
0.14
0.16
0.18
0.2
Vietrios-Rips (dimension 1)
Vietoris-Rips (dimension 2)
Components
Filtration parameter ϵ

B3

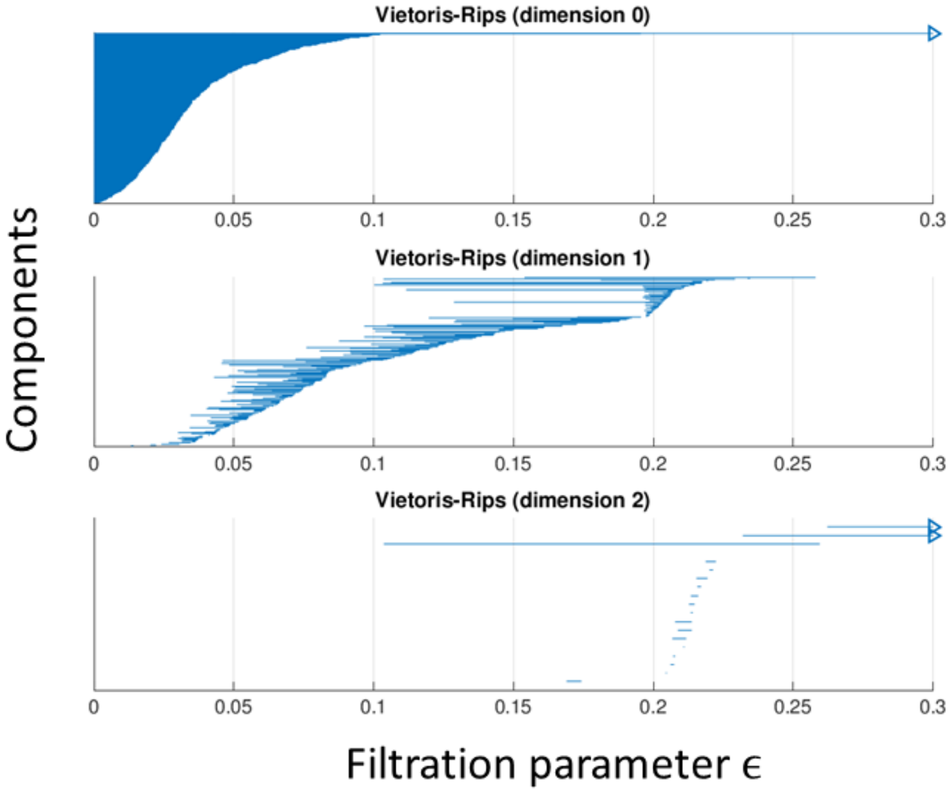
Vietoris-Rips (dimension 0)
Vietoris-Rips (dimension 1)
Vietoris-Rips (dimension 2)
0
0.05
0.1
0.15
0.2
0.25
0.3
Components
Filtration parameter ϵ

B4

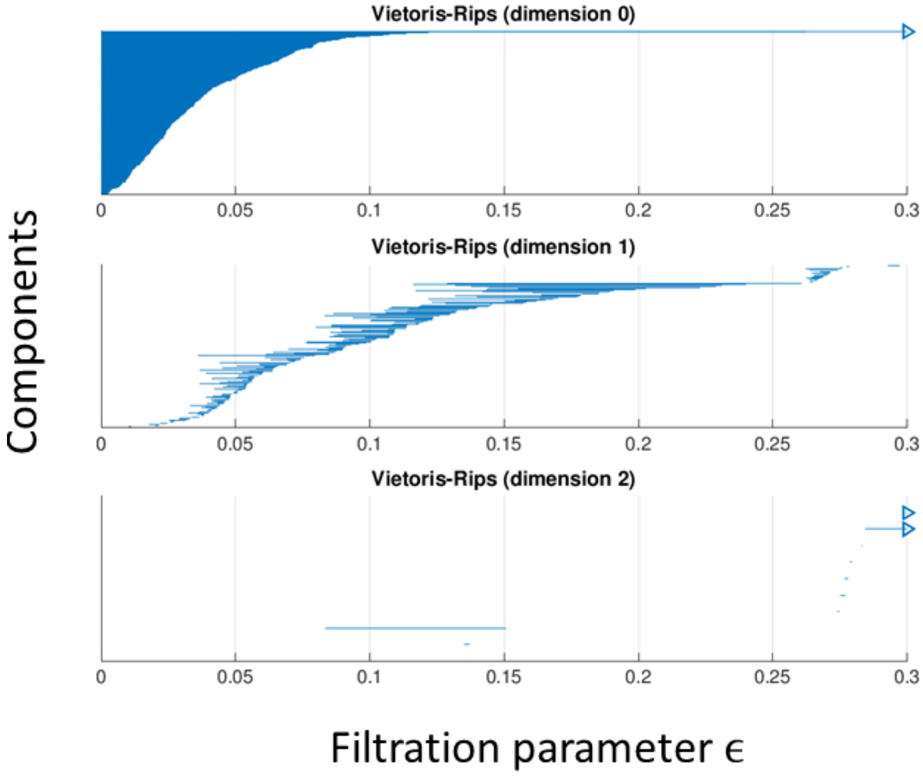
Vietoris-Rips (dimension 0)
Vietoris-Rips (dimension 1)
Vietoris-Rips (dimension 2)
0
0.05
0.1
0.15
0.2
0.25
0.3
Components
Filtration parameter ϵ

C1

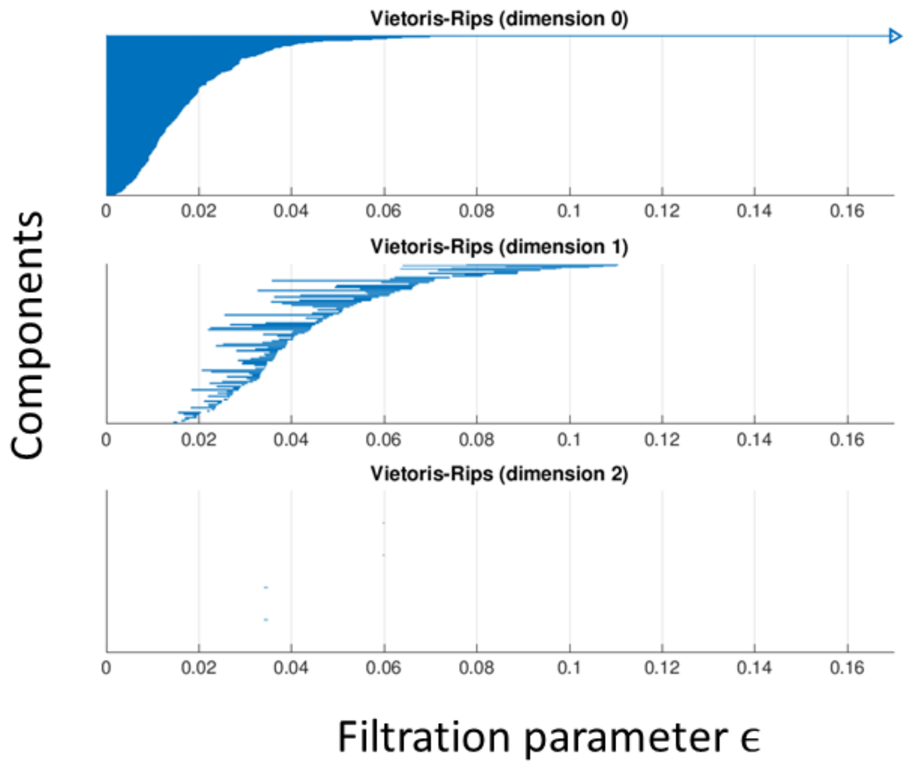

Vietoris-Rips (dimension 0)
Vietoris-Rips (dimension 1)
Vietoris-Rips (dimension 2)
0
0.02
0.04
0.06
0.08
0.1
0.12
0.14
0.16
Components
Filtration parameter ϵ

C2

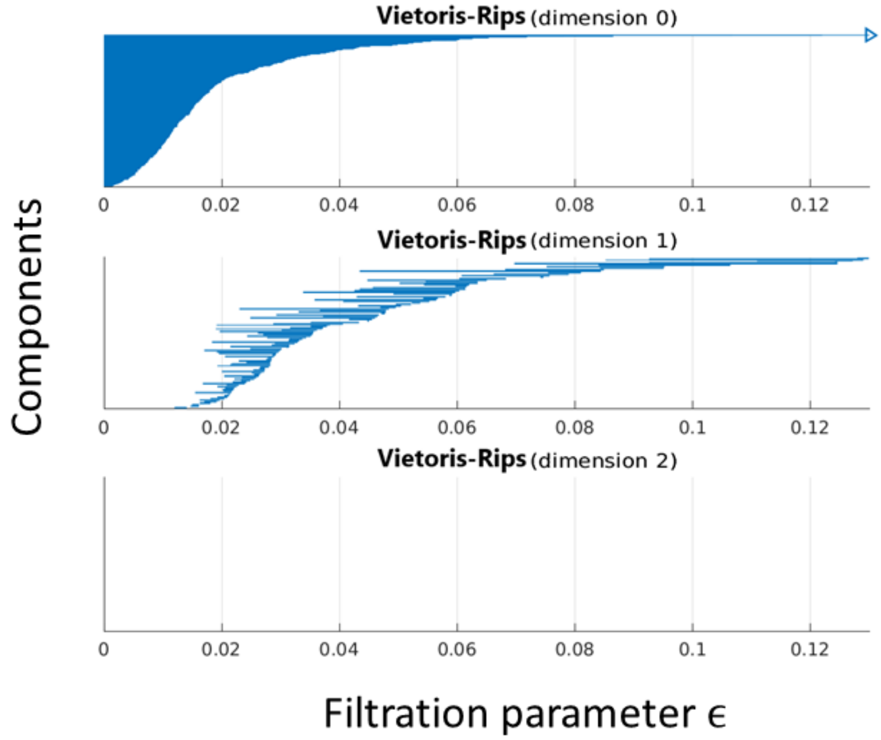

Vietoris-Rips (dimension 0)
Vietoris-Rips (dimension 1)
Vietoris-Rips (dimension 2)
0
0.02
0.04
0.06
0.08
0.1
0.12
Components
Filtration parameter ϵ

C3

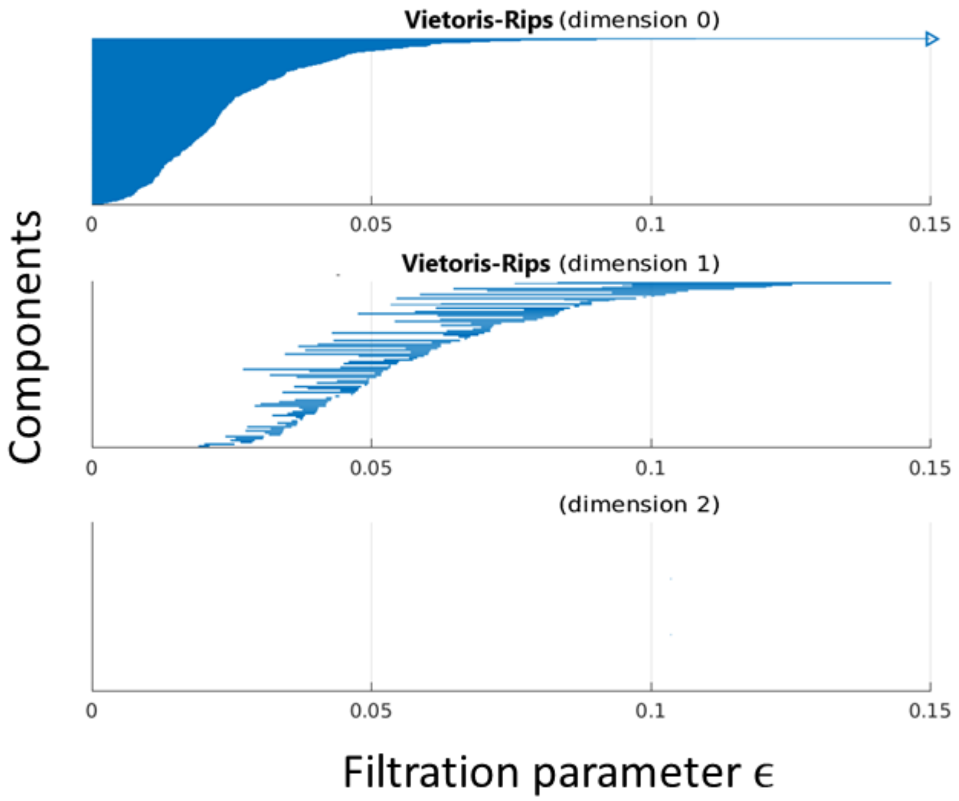

Vietoris-Rips (dimension 0)
Vietoris-Rips (dimension 1)
(dimension 2)
0
0.05
0.1
0.15
Components
Filtration parameter ϵ

C4

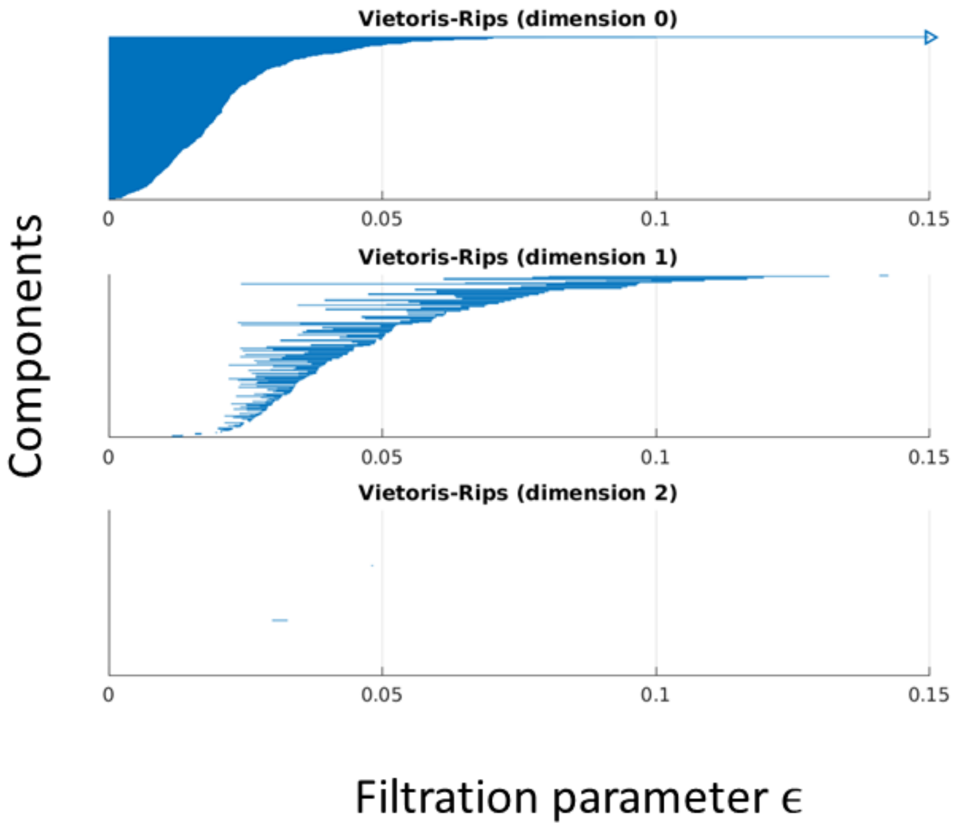

Vietoris-Rips (dimension 0)
Vietoris-Rips (dimension 1)
Vietoris-Rips (dimension 2)
0
0.05
0.1
0.15
Components
Filtration parameter ϵ

D1

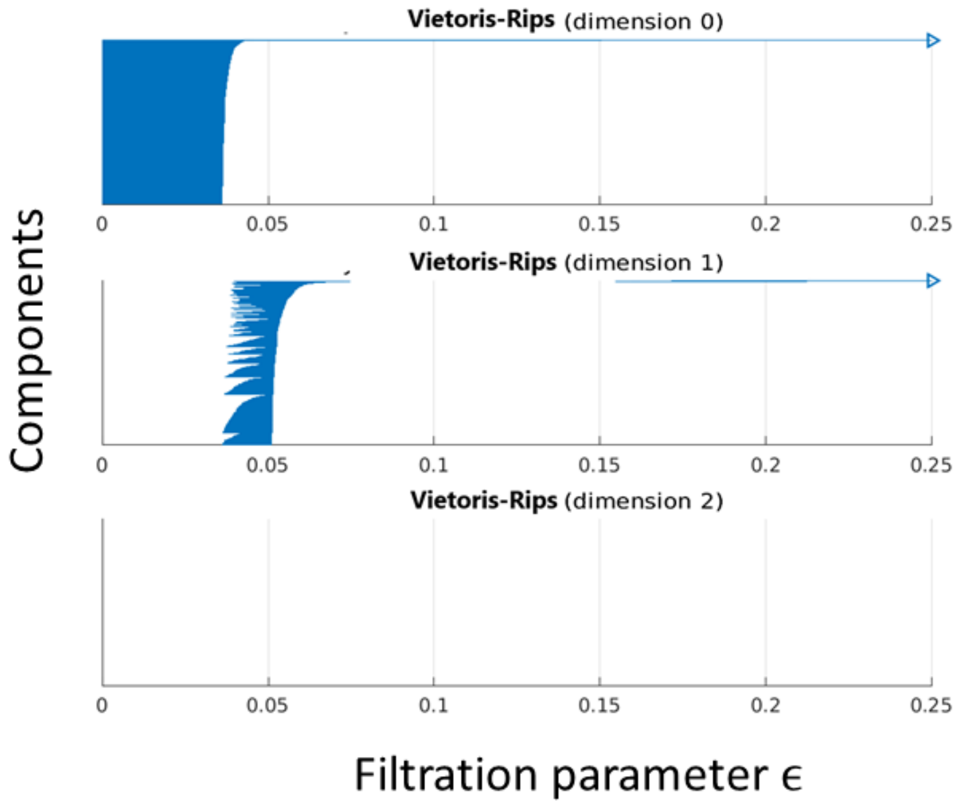
Vietoris-Rips (dimension 0)
Vietoris-Rips (dimension 1)
Vietoris-Rips (dimension 2)
0 0.05 0.1 0.15 0.2 0.25
Components
Filtration parameter ϵ

D2

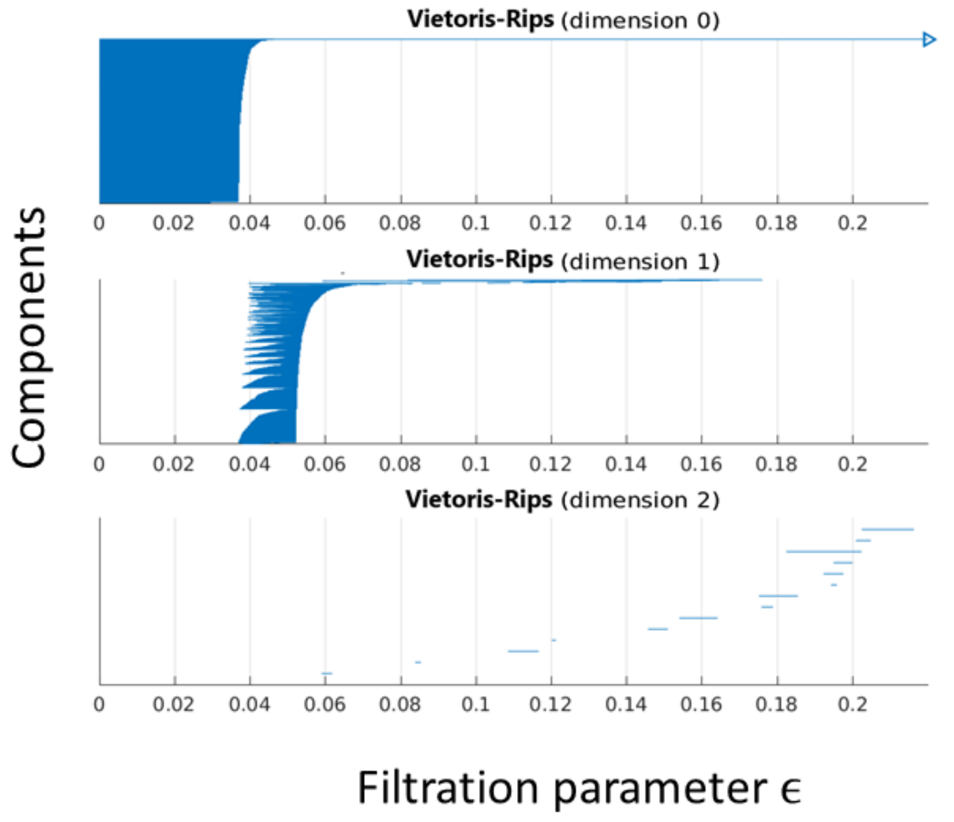
Vietoris-Rips (dimension 0)
Vietoris-Rips (dimension 1)
Vietoris-Rips (dimension 2)
0 0.02 0.04 0.06 0.08 0.1 0.12 0.14 0.16 0.18 0.2
Components
Filtration parameter ϵ

D3

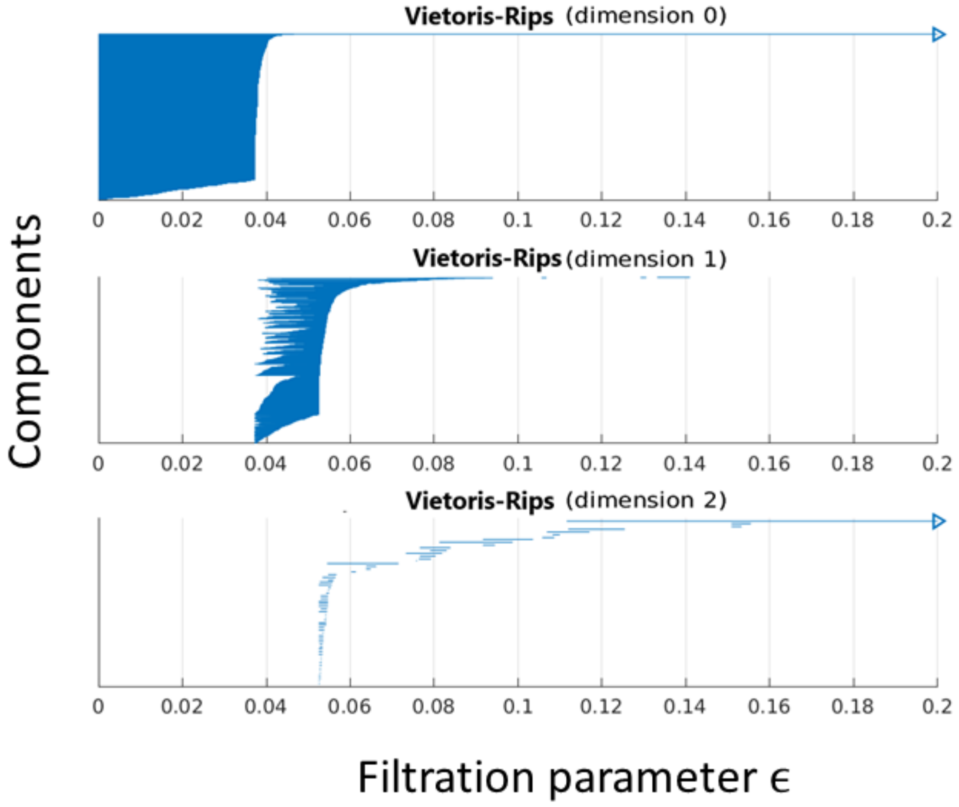
Vietoris-Rips (dimension 0)
0 0.02 0.04 0.06 0.08 0.1 0.12 0.14 0.16 0.18 0.2
Vietoris-Rips (dimension 1)
0 0.02 0.04 0.06 0.08 0.1 0.12 0.14 0.16 0.18 0.2
Vietoris-Rips (dimension 2)
0 0.02 0.04 0.06 0.08 0.1 0.12 0.14 0.16 0.18 0.2
Components
Filtration parameter ϵ

D4

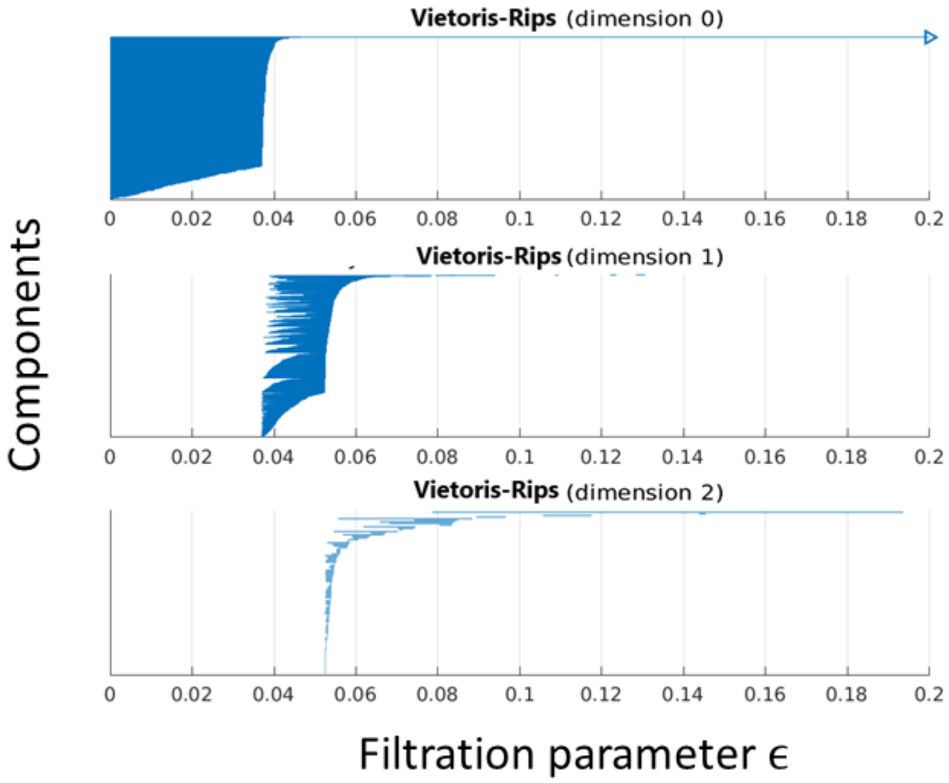
Vietoris-Rips (dimension 0)
0 0.02 0.04 0.06 0.08 0.1 0.12 0.14 0.16 0.18 0.2
Vietoris-Rips (dimension 1)
0 0.02 0.04 0.06 0.08 0.1 0.12 0.14 0.16 0.18 0.2
Vietoris-Rips (dimension 2)
0 0.02 0.04 0.06 0.08 0.1 0.12 0.14 0.16 0.18 0.2
Components
Filtration parameter ϵ

Supplementary Figure S1. The barcodes of the 16 fusion mechanisms.

01082019 - 31012020

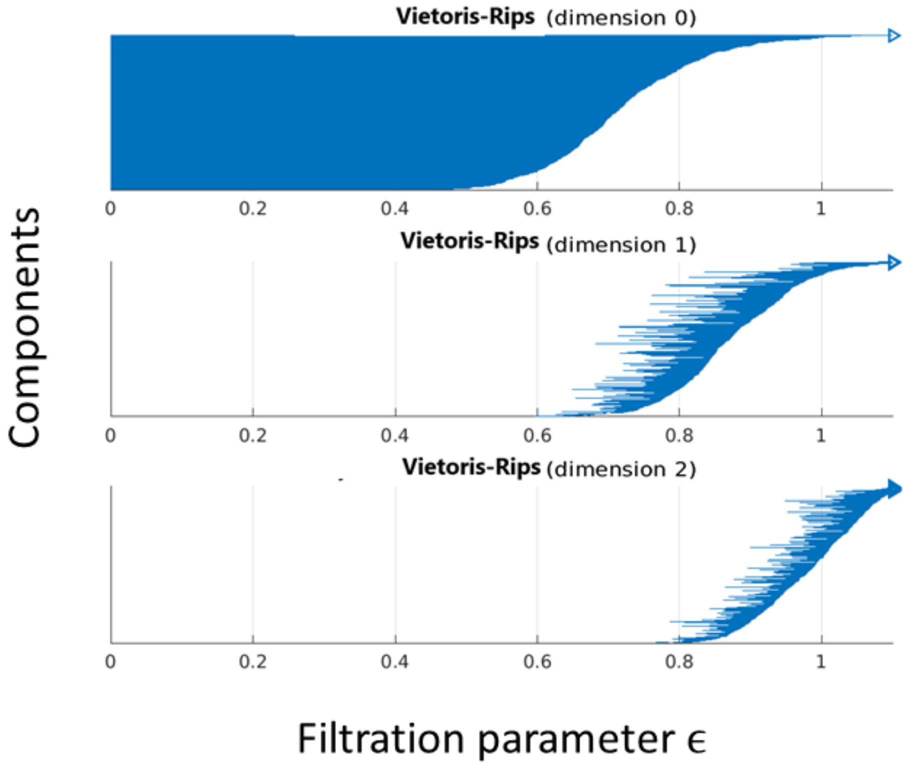

08082019 - 08022020

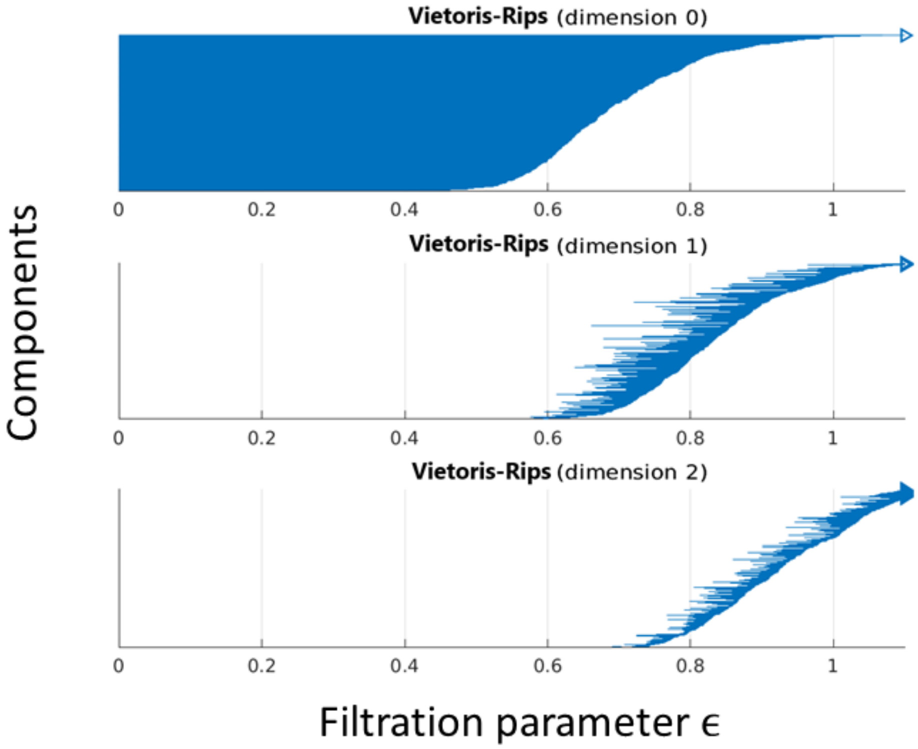

15082019 –15022020

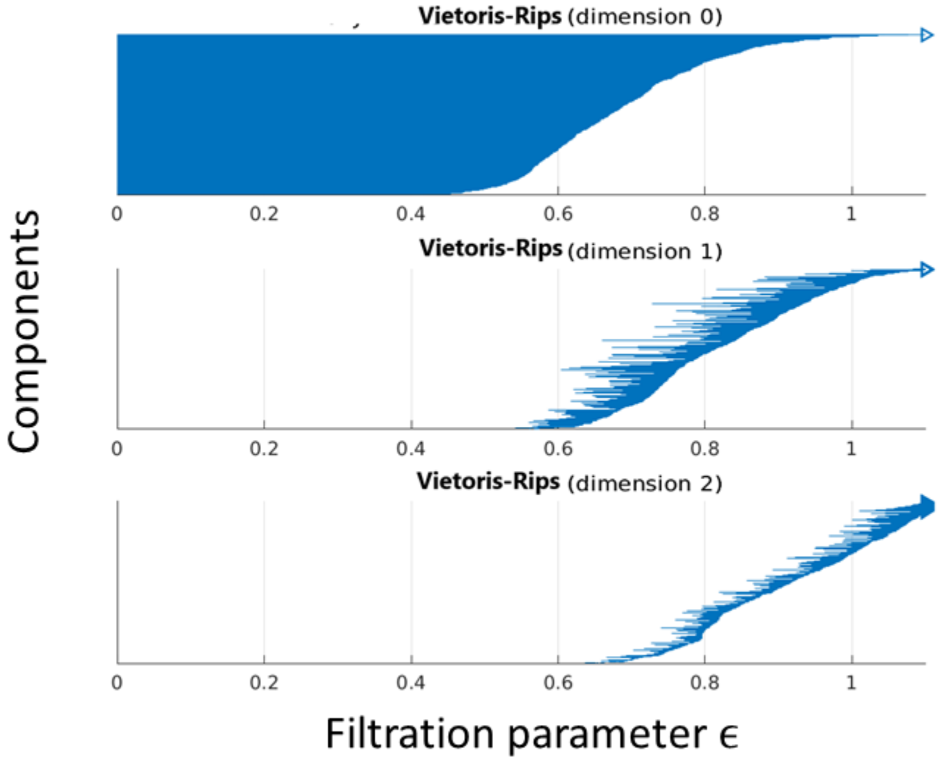

22082019 –22022020

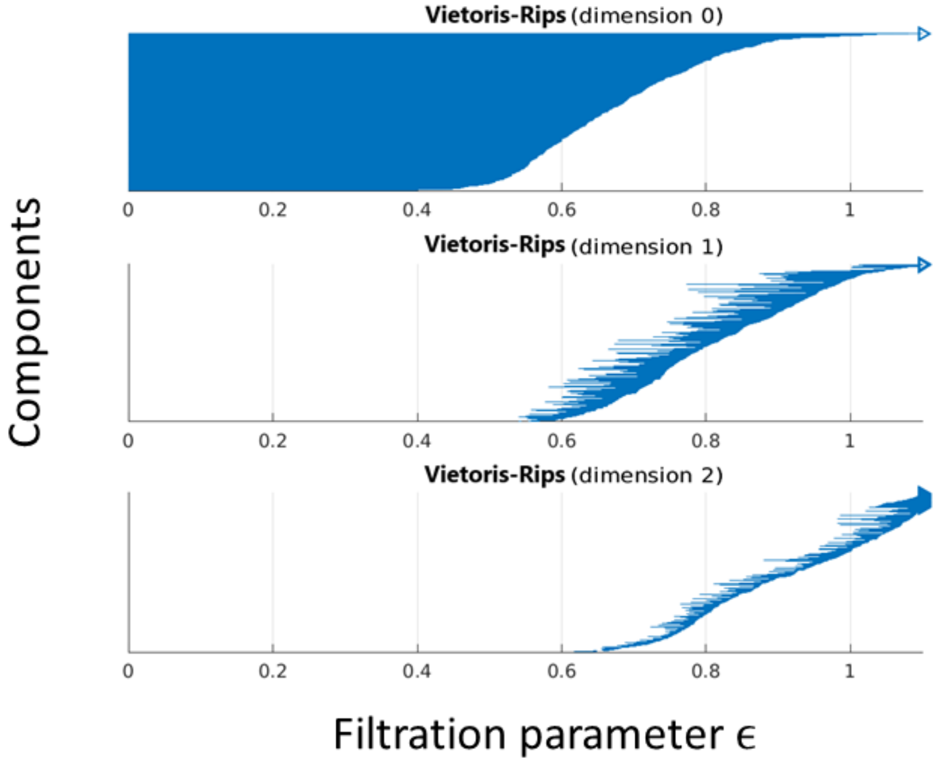

01092019 –01032020

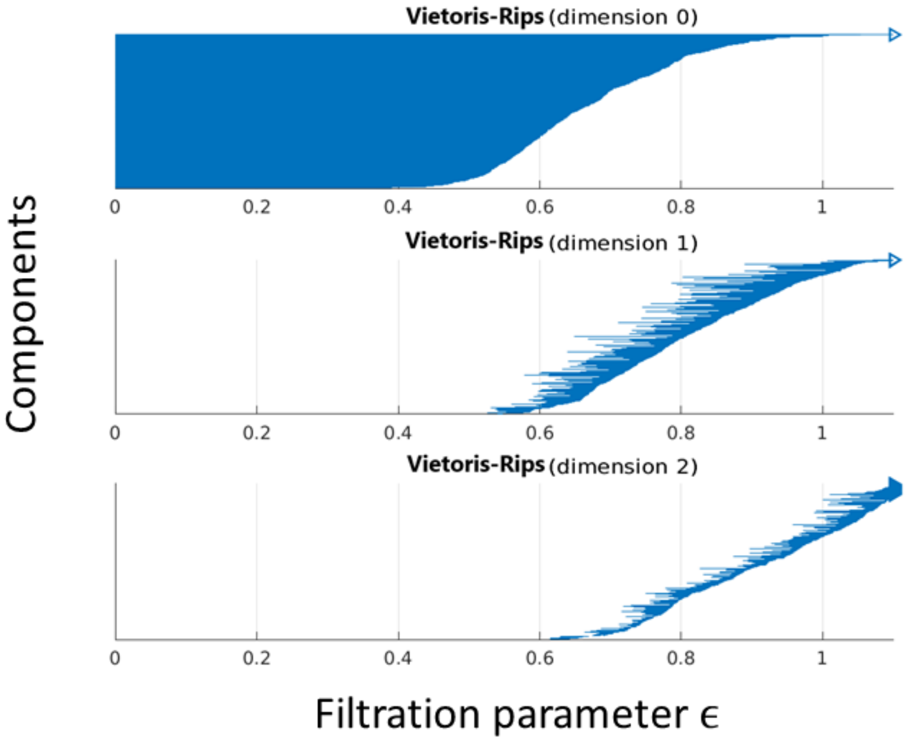

08092019 –08032020

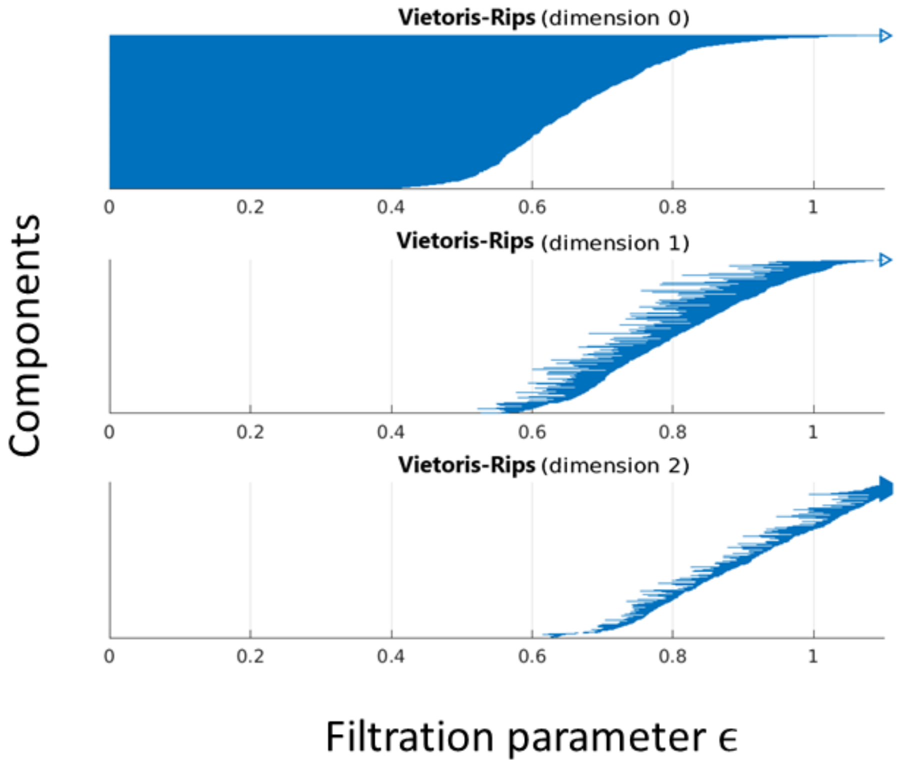

15092019 –15032020

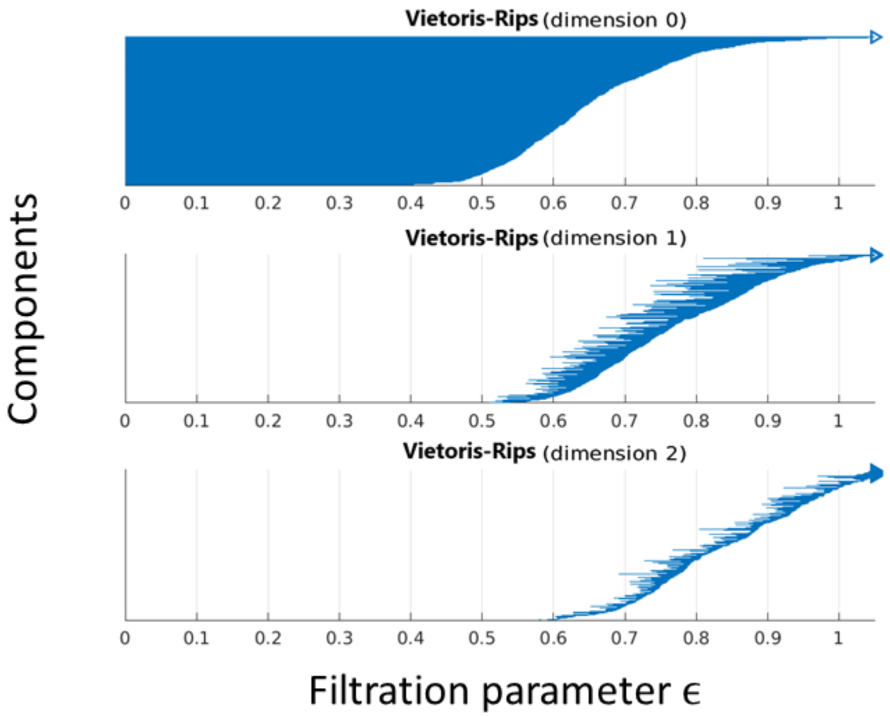

22092019 –22032020

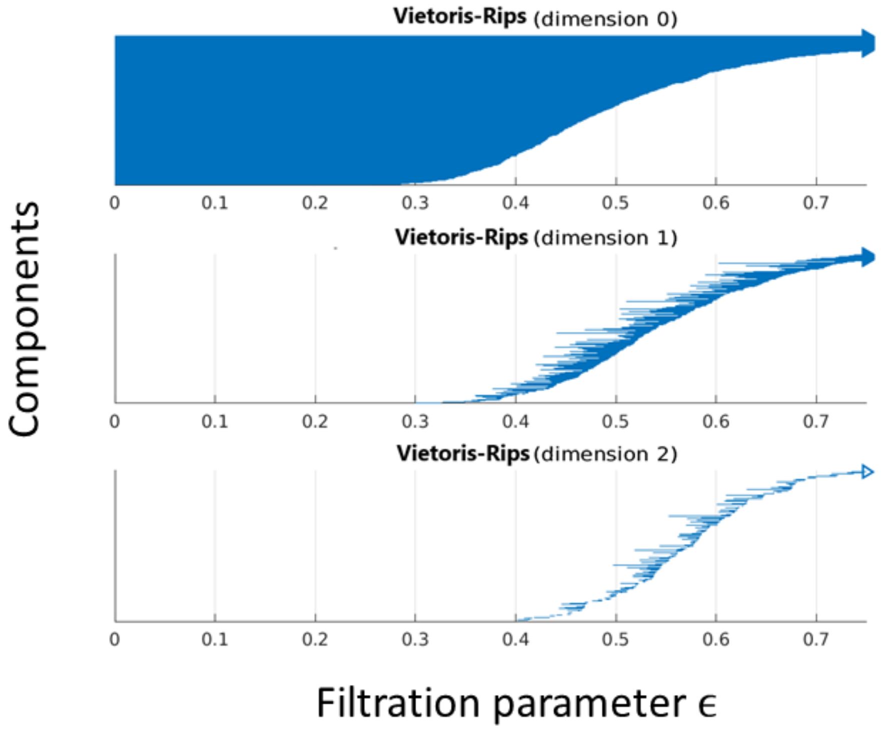

01102019 –31032020

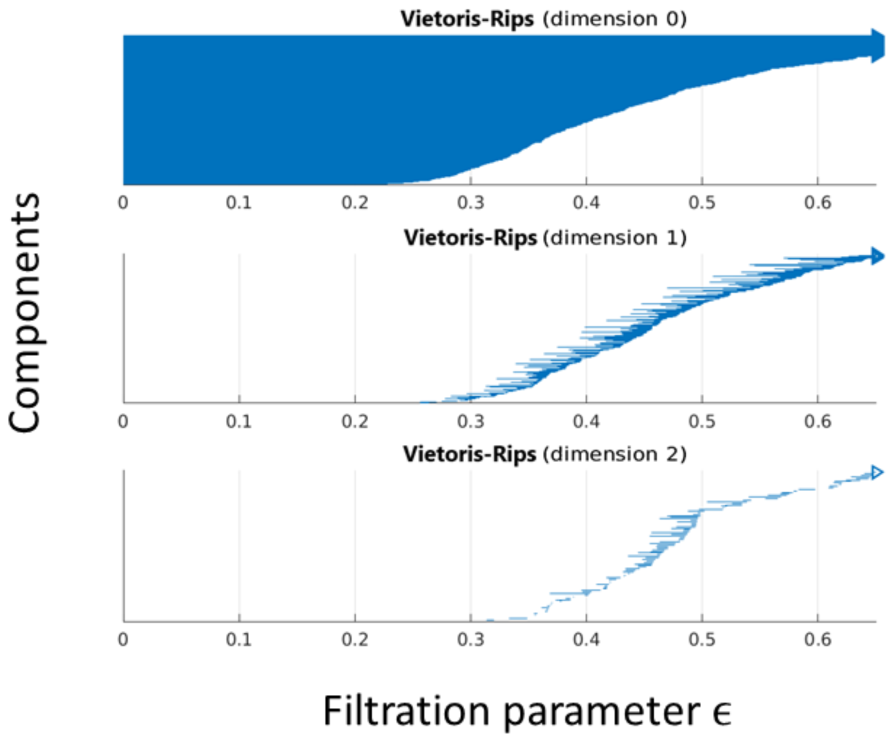

Supplementary Figure S2. The barcodes of the nine periods from 1 August 2019 to 31 March 2020 of TWSE.
